# Differences Between Health- and Skill-Related Physical Fitness Profiles of Kenyan Children from Urban and Rural Areas: The Kenya-LINX Project

**DOI:** 10.3390/ijerph22040542

**Published:** 2025-04-02

**Authors:** Stanley Kagunda Kinuthia, Gareth Stratton, Lucy Joy Wachira, Victor Okoth, George Evans Owino, Sophie Ochola, Amie Bethan Richards, Festus Kiplamai, Vincent Onywera, Nils Swindell

**Affiliations:** 1 Department of Physical Education, Exercise and Sports Science, Kenyatta University, Nairobi P.O. Box 43844-00100, Kenya; wachira.lucy@ku.ac.ke (L.J.W.); kiplamai.festus@ku.ac.ke (F.K.); 2 Applied Sports, Technology, Exercise and Medicine (A-STEM) Research Centre, Swansea University, Swansea SA1 8EN, UK; g.stratton@swansea.ac.uk (G.S.); amie.richards@swansea.ac.uk (A.B.R.); n.j.swindell@swansea.ac.uk (N.S.); 3 Department of Environmental Science and Education, Kenyatta University, Nairobi P.O. Box 43844-00100, Kenya; victorokoth44@gmail.com; 4 African Population and Health Research Centre, Kitisuru, Manga Close, Kirawa Road, Nairobi P.O. Box 10787-00100, Kenya; gowino@aphrc.org; 5 Department of Food, Nutrition and Dietetics, Kenyatta University, Nairobi P.O. Box 43844-00100, Kenya; ochola.sophie@ku.ac.ke; 6 Division of Research, Innovation and Outreach, KCA University, Nairobi P.O. Box 56808-00200, Kenya; vonywera@kcau.ac.ke

**Keywords:** body mass index, cardiorespiratory fitness, health disparities, physical fitness

## Abstract

Physical fitness is a key indicator of children’s health, yet amidst rising inactivity and obesity, data on Kenyan children are scarce. This study assessed health- and skill-related fitness differences between rural and urban Kenyan children while examining demographic influences. Cardiorespiratory fitness (CRF), BMI, strength, flexibility, speed, agility, and coordination were assessed in 1131 children aged 11.07 ± 0.9 years (52.7% girls) recruited using stratified cluster random sampling. Significant rural–urban disparities were observed. In urban areas, 16.6% were overweight and 2.8% obese, compared to 4% and 0.6% in rural areas (*p* < 0.001). Conversely, 44.5% of the rural cohort were underweight versus 13.7% urban cohort (*p* < 0.001). Multivariable regression revealed that rural children demonstrated superior CRF (β = −4.68 laps, *p* < 0.001) and lower back flexibility (β = −2.77 cm, *p* < 0.001), while urban children excelled in speed and coordination (β = 3.68 bounces, *p* < 0.001) and grip strength (β = 2.16 kg, *p* < 0.001). Boys outperformed girls in explosive leg power (β = −6.75 cm, *p* < 0.001) and CRF (β = −6.92 laps, *p* < 0.001). These findings highlight fitness inequities among Kenyan children, emphasising the need for equitable, targeted, and inclusive physical activity opportunities.

## 1. Introduction

Physical fitness (PF) is a marker for healthy growth and development in children, influencing their physical, cognitive, and emotional well-being [1,2,3]. Early development of PF lays the foundation for a healthy lifestyle and impacts long-term health trajectories and overall quality of life, as poor PF increases vulnerability to obesity and cardiovascular issues [4]. Regular physical activity (PA) enhances cardiovascular health, muscular strength, and body composition [5,6] while reducing inflammation and improving insulin sensitivity [7]. It lowers the risk of metabolic and cardiovascular diseases, type 2 diabetes, and positively impacts mental health by alleviating stress and anxiety [8]. Additionally, improved fitness is associated with better cognitive function, which is essential for academic success [9].

The decline in PF has serious long-term health implications, increasing the risk of chronic diseases and negatively affecting academic performance and cognitive function. Low fitness levels can diminish self-esteem and social interactions. Therefore, early targeted interventions are crucial to promote healthier lifestyles and enhance children’s overall quality of life in both urban and rural settings [10,11].

However, children’s overall fitness, including their muscular and cardiorespiratory fitness (CRF), has declined in recent years [12]. The noted decrease in flexibility, strength, and speed among adolescents and school-aged children is also a concern [13,14]. This decline may be due to demographic changes and reduced PA [12].

Globally, declining PF levels among children, especially in high-income countries, are linked to increased sedentary behaviours and dietary changes [11,12,15]. In low- and middle-income countries like Kenya, rapid urbanisation and socioeconomic shifts have created a dual burden of undernutrition and overnutrition among children [16,17]. In Sub-Saharan Africa, including Kenya, there have been rapid social, economic, and urban changes [18,19]. Unfortunately, this has also led to a rise in inactivity and obesity, with half of Kenyan children not meeting international PA guidelines, 21% of children in Nairobi being overweight or obese [18,20], and only 7% complying with the combined 24 h movement guidelines [21]. Furthermore, health inequalities are evident; for example, obesity and insufficient PA are more frequent among girls than boys in rural Kenya [18,22]

Research indicates that urban children often have greater access to organised sports and recreational facilities, which contributes to structured PA. However, they also exhibit higher rates of sedentary behaviour and obesity due to increased screen time and processed food consumption. Rural children, despite facing challenges such as undernutrition and limited resources, often engage in a variety of unstructured physical activities that vary significantly in intensity and type [23,24,25,26,27].

However, existing data predominantly represent high-income and upper-middle-income countries, leaving a gap in knowledge for low-middle-income countries [28]. Though data are sparse in Sub-Saharan Africa, they suggest that PF is declining among children in Kenya [20,29]. Currently, there are limited data from Sub-Saharan Africa, particularly Kenya, where urban–rural dynamics significantly affect health outcomes [16].

International standards have classified school children as adequately fit or not, and thus in need of interventions to improve their health [30,31,32]. However, Kenya needs a specific strategy for monitoring PF in children, and there is a need for more comprehensive and representative data on this topic as established in developed countries [33,34,35]. To our knowledge, few studies have investigated PF in Kenyan school children [25,36]. However, these studies were limited by very small samples and did not account for urban and rural differences [25,36].

Age, gender, body mass index (BMI), education, genetic predisposition, and the environment can all influence a child’s PF levels [37,38,39,40]. Such correlates should be considered when designing programmes to improve PF among children.

The limited available evidence on the health- and skill-related fitness of Kenyan school children is also dated and overlooks urban–rural challenges, including rapid urbanisation and lifestyle shifts over the past decade, which have driven health disparities. To support decision-making on appropriate interventions and establish ongoing surveillance for addressing potential public health issues, further research is needed to collect comprehensive data on children’s health and fitness levels across multiple schools in both urban and rural areas.

This study addresses the urgent need to understand health- and skill-related fitness among Kenyan children amidst these rapid changes. By comparing urban and rural populations, it aims to identify health and fitness disparities and provide representative data to inform targeted public health strategies and interventions against rising inactivity and obesity. Therefore, this study aimed to assess the differences in health- and skill-related fitness between children from rural and urban Kenya. The findings will guide targeted interventions and influence policy decisions and educational practices, promoting equitable access to physical activity opportunities and improving health outcomes in Kenya. This is particularly important as childhood fitness gains recognition in combating non-communicable diseases, supporting long-term monitoring of children’s health.

## 2. Materials and Methods

### 2.1. Sample and Study Design

This study was part of a larger Kenyan-based research project, the “Kenya Healthy Diet and Active Lifestyle Infrastructure” for the NeXt Generation (Kenya-LINX), to understand the needs of children and schools to improve their health and well-being. This cross-sectional study involved 1131 children aged 9–11 years from Nairobi City and Kitui counties in Kenya. The study received the necessary permits and authorisation from the National Commission for Science, Technology, and Innovation (NACOSTI) [License No: NACOSTI/P/20/5030]. The authority to conduct the study was obtained from County and Sub-County Education offices in Nairobi and Kitui Counties, and permission to access the school was sought from school heads.

### 2.2. School and Participant Recruitment

Children from 17 schools in urban county (Nairobi City: *n* = 650, 53.2% female, 10.97 ± 0.83 years) and 16 schools in rural county (Kitui: *n* = 481, 51.8% female, 11.19 ± 0.95 years) in Kenya were recruited for the study. Stratified cluster random sampling was employed to ensure representation across geographical settings and school types, consistent with established guidelines for epidemiological studies [41]. Initially, a comprehensive list of all primary schools within the selected sub-counties of Nairobi City and Kitui was compiled. Schools were then stratified based on their location (urban/rural) and type (public/private). Within each stratum, schools were randomly selected using a computerised random number generator following procedures outlined by Lim and colleagues [42]. As a result, ten private and seven public schools in Nairobi City County and seven private and nine public schools in Kitui County were selected from across three sub-counties in each respective county. Subsequently, within each selected school, one class from each of grades 3, 4, and 5 was randomly selected, and all children within those classes were invited to participate. This multi-stage approach aimed to minimise selection bias and ensure a representative sample [41]. Only children in grades 3–5 from a particular class in each school were included, and those who declined or were under-age or over-age were excluded. The study did not include boarding and single-sex schools because they do not represent significant Kenyan primary school attendance. Excluding these schools potentially limits the study’s generalisability. Boarding schools, with their structured routines, might have shown higher fitness levels, while single-sex schools could exhibit gender-specific fitness profiles due to varied activity focus. The study details were fully explained to the schools, children, and parents (where necessary, in their native mother tongue), and those who agreed to participate signed the consent/assent forms. Data collection occurred during school hours on the school premises.

### 2.3. Testing Procedures and Instruments

Standardised fitness assessments as previously reported through ‘fitness fun days’ [33,34] were administered following procedures outlined in the Kinanthropometry and Exercise Physiology Laboratory Manual. The chosen tests were reliable, feasible for use in schools, and proven valid [43]. Prior to testing, all equipment was calibrated, and testing areas were flat and clearly marked. To ensure consistency, the same equipment was used across all schools, and evaluators (PE, exercise, and sports science professionals) underwent training sessions to standardise measurement techniques. Before each test, participants received demonstrations and verbal instructions, and were allowed practice trials to familiarise themselves with procedures and minimise the learning effect. The standardised testing sequence began with anthropometric measurements, followed by speed bounce, sit-and-reach, grip strength, 5 × 10 m shuttle run, standing broad jump, and 20 m MSFT. This order was maintained to minimise variability. Each test was performed in duplicate or triplicate, and the highest or average score was used for analysis. Participants were also given breaks between each test to mitigate fatigue. The tests were conducted in small groups at agreed-upon times in PE halls and school fields. To minimise potential bias, evaluators were blinded to the study’s hypotheses and the urban or rural classification of participants during data collection. All evaluators were trained using standardised protocols, ensuring consistency in test administration and scoring. Data entry was conducted using a digital platform with built-in validation checks to reduce errors. To minimise subjective bias, evaluators did not have access to the participants’ personal information during testing. Furthermore, data entry was cross-verified by the principal researchers to reduce errors and bias.

#### 2.3.1. Components of Health-Related Fitness

The health-related measures included CRF, strength, BMI, and flexibility. CRF was measured using the 20 m multistage fitness test (20 m MSFT) that followed the protocol established by Leger et al. [44]. The scoring criterion was based on the total number of laps completed without missing the beep or upon reaching exhaustion. The children’s hand grip strength was measured using a hand grip dynamometer with an adjustable grip (Takei 5001; Takey, Tokyo, Japan) while standing with their arms straight by their sides. The children’s anthropometric measures of height and weight were collected using a portable SECA 214 standing stature stadiometer and a SECA 876 digital scale (SECA, Hamburg, Germany). Two measurements were taken for each, with a third measurement taken if the difference between the first two measurements was more than 0.5 cm (cm) for height and 0.1 kg (kg) for weight. The BMI of each participant was calculated using weight and height measurements. BMI z-scores were then computed using the LMS method, which accounts for age and sex-specific growth patterns. This method utilised the L (skewness), M (median), and S (coefficient of variation) parameters from the WHO reference population to standardise BMI values. Based on the International Obesity Task Force (IOTF) cut-offs, participants were categorized as below a healthy weight, healthy weight, overweight, or obese [45]. The participants were tested for flexibility using the sit and reach test (SAR) protocol.

#### 2.3.2. Components of Skill-Related Fitness

Children performed the speed bounce test (speed, endurance, and coordination), 10 × 5 m shuttle run test (5 × 10 m SRT) (running speed and agility), and standing broad jump test (SBJ) (explosive leg power). In the speed bounce test, children performed two trials with a 10 s break in between, and the highest score of the 2 trials was used for analysis. The 5 × 10 m SRT involved participants running back and forth between markers covering a total distance of 50 m. They had to cross both markers with both feet, and the fastest time from the 2 trials was recorded in s. The standing broad jump mat was used to measure distance. Children jumped forward, landing on both feet and using their arms and legs to propel themselves forward as far as possible without falling backward. They were given 3 trials, and the average of their scores was calculated in centimetres (cm).

#### 2.3.3. Data Collection and Quality Control

The study utilized the ESRI ArcGIS Survey 123 app for data collection. The evaluators accessed the data entry templates virtually using tablets and entered the data in the app, which translated the scores into a Microsoft Excel spreadsheet. The evaluators also recorded hard copy backup test results to verify the entered data and check for accuracy. The lead author reviewed the data to ensure accuracy. The online data entry platform simplified data collection, management, and quality control since the online protocols automatically created a unified view of all the data assets ready for migration.

### 2.4. Statistical Analysis

A priori power analysis using G*Power determined that a minimum of 880 participants were needed to detect differences in fitness between urban and rural children, based on an effect size of 0.3, significance level of 0.05, and power of 0.80. The final sample of 1131 children surpassed the required threshold, ensuring sufficient statistical power for the analyses. The mean and standard deviation (SD) were calculated for all continuous variables, whereas categorical variables were presented as absolute values (n) and percentages (%). Independent samples *t*-tests (t) were utilised to compare continuous variables, while Chi-square tests (χ^2^) were applied for categorical variables. Significant associations were followed up with pairwise comparisons using a χ^2^ post hoc test, and Bonferroni adjusted *p*-values to account for multiple comparisons [46]. A multi-level model was employed to examine associations between demographics and fitness measures while accounting for the clustering of children within schools, treating school as a random effect and incorporating age, gender, area, and weight status as fixed factors [47]. Before conducting multivariable regression, multicollinearity among predictor variables was assessed using variance inflation factor (VIF). A VIF of <4 confirmed that multicollinearity was not a concern. Statistical significance was set at *p* < 0.05, and analyses were conducted using IBM Statistical Package for the Social Sciences (SPSS) statistics version 28 for Windows.

## 3. Results

The participants’ characteristics are presented in Table 1. A total of 1131 children aged 9 to 11 years (52.6% female) were recruited: 481 children from rural areas and 650 from urban areas. Of all participants, 51.5% attended public schools, and 48.5% attended private schools. On average, the children were 11.07 years old, with rural children slightly older (11.19 years) than urban children (10.97 years) (*p* < 0.001). The age difference between rural and urban children had a small effect size (d = 0.25). A high proportion of children from both urban and rural schools had healthy weight, with at least half of rural children (50.9%) and two-thirds of urban children (66.9%) falling into this category (*p* < 0.001); 44.5% of rural and 13.7% of urban children were below healthy weight (*p* < 0.001).

Table 2 displays the percentage of children who achieved the fitness thresholds for CRF, explosive leg power, and arm strength. A greater proportion of children from rural areas (22.7%) met the CRF threshold compared to children from urban areas (8.4%). On the other hand, a higher proportion of children from urban areas (86.8%) met the arm strength threshold compared to children from rural areas (66.5%). Over 75% of children achieved the required threshold for explosive leg power, with children from rural areas having a higher success rate (79.4%) than children from urban areas (75.3%). The χ^2^ test indicated significant differences between children from rural and urban areas reaching and not reaching the threshold for CRF (χ^2^ = 45.10, df = 1, *p* < 0.001) and arm strength (χ^2^ = 66.57, df = 1, *p* < 0.001). χ^2^ post hoc tests showed significant differences between children from rural and urban areas achieving the thresholds for CRF (*p* < 0.001) and arm strength (*p* < 0.001).

Table 3 presents the results of the multi-level model analysis. After controlling for differences in sex, area, age, and weight status, children from urban areas scored higher in speed, endurance, and coordination, hand grip strength, running speed and agility, and explosive leg power than children from rural areas. However, children from rural areas exhibited superior lower back flexibility and CRF.

Children from urban areas demonstrated superior performance in speed, endurance, coordination (β = 3.68, *p* < 0.001), explosive leg power (β = 2.25, *p* = 0.046), and hand grip strength (β = 2.16, *p* < 0.001). In contrast, rural children outperformed urban children in lower back flexibility (β = −2.77, *p* < 0.001) and CRF (β = −4.67, *p* < 0.001).

Age-related trends indicated that older children exhibited improved performance across most fitness variables, including speed, endurance, coordination (β = 1.60, *p* < 0.001), hand grip strength (β = 1.39, *p* < 0.001), and explosive leg power (β = 2.23, *p* < 0.001).

Gender differences were also evident, with boys consistently outperforming girls in explosive leg power (β = −6.75, *p* < 0.001), CRF (β = −6.92, *p* < 0.001), and running speed and agility (β = 0.49, *p* < 0.001). However, girls demonstrated slightly better lower back flexibility in urban areas, as indicated by a significant interaction effect (β = 1.83, p = 0.023).

Being overweight and obese was negatively associated with performance in speed, endurance, and coordination (β = −5.83, *p* < 0.001; β = −5.24, *p* = 0.001), explosive leg power (β = −12.22, *p* < 0.001; β = −12.65, p = 0.001), and running speed and agility (β = 0.72, *p* < 0.001; β = 0.92, *p* = 0.036). Additionally, being overweight was negatively associated with CRF (β = −5.03, *p* < 0.001) but positively associated with hand grip strength (β = 1.44, *p* < 0.001). Conversely, being below a healthy weight was negatively associated with hand grip strength (β = −1.98, *p* < 0.001) and positively associated with CRF (β = 3.09, *p* < 0.001).

The univariate regression coefficients are generally larger than the multivariate ones, indicating some confounding effects. For example, the effect of area (urban) on speed, endurance, and coordination decreased from β = 4.12 (univariate) to β = 3.68 (multivariate). The effect of age on hand grip strength decreased from β = 1.65 (univariate) to β = 1.39 (multivariate).

## 4. Discussion

The aim of this study was to compare the health- and skill-related fitness of school-going children from urban–rural areas of Kenya, laying the groundwork for future research on PF in children, adolescents, and youth of various ages. The findings revealed that urban children had higher rates of overweight and obesity, along with superior performance in speed and coordination and handgrip strength. In contrast, rural children exhibited higher rates of underweight, stronger CRF, and flexibility. Additionally, boys consistently outperformed girls in various fitness components, while overweight children exhibited stronger handgrip strength compared to their underweight and obese peers.

The calculated effect sizes (Cohen’s d) emphasise the practical significance of findings, showing substantial impacts of environmental factors on children’s health, particularly in body mass, BMI, and stature. This suggests that urban children likely benefit from better nutrition and healthcare access. Cohen’s d indicate that the disparities in fitness outcomes are not only statistically significant but also meaningful. For example, a large Cohen’s d value for the differences in speed and agility between urban and rural children underscores the need for targeted interventions to address these gaps.

Moreover, univariate analyses reveal that urban children consistently show lower performance in various fitness outcomes compared to their rural peers. This highlights unique challenges in urban environments, such as limited access to safe recreational spaces and increased sedentary behaviours, which may contribute to poorer fitness levels. The univariate coefficients indicate that while urban children may excel in certain areas, such as handgrip strength, they lag in others, reinforcing the need for comprehensive strategies to promote healthier lifestyles. These findings are consistent with recent studies from South Africa [48], the United States [49], and Kenya [26,36].

The differences in PF between boys and girls warrant further exploration. Biological factors, such as muscle mass development and hormonal influences, may explain boys’ superior performance in fitness tests [50]. Sociocultural factors, including societal expectations and resource access, can restrict girls’ participation in physical activities, potentially contributing to the observed differences in fitness levels, especially in urban areas [51,52].

Socioeconomic disparities significantly impact PF outcomes. Urban families may have higher incomes but less time for PA due to work demands, while rural families prioritise physical labour [53]. In addition, urban schools often lack safe recreational spaces, whereas rural schools have more open areas but fewer structured programmes. These factors collectively contribute to fitness disparities [54].

The results indicate that a significantly greater proportion of children from urban areas were overweight, while in rural areas, significantly more children were underweight, emphasising the contrasting public health challenges faced in Kenya. While this study did not focus on nutrition or socioeconomic factors, they may contribute significantly to the observed disparities. Research shows that urban children consume more processed foods, whereas rural children experience food insecurity, resulting in undernutrition [55,56]. Furthermore, rural children had higher flexibility and aerobic fitness but lower speed, agility, coordination, leg power, and hand grip strength. These findings extend results from a smaller study conducted in Kenya [25] and concur with a South African study [57] that shows that children from urban areas have lower levels of PF performance than their rural peers.

Children from rural areas have poorer performance in running speed and agility. This can be attributed to limited access to structured PA programmes, sports facilities, and proper equipment, while urban schools in Kenya provide better facilities, coaching, and organised training. These disparities necessitate further research [21,36,58].

The inequities in the health-and skill-related PF of children from urban and rural Kenya suggests that a community’s environment and physical activities can impact children’s behaviour and socialisation. Schools may have different challenges in promoting fitness depending on the opportunities available in the community [59]. Fewer children from urban areas met the combined movement guidelines [21] due to limited space, safety concerns, and high crime rates [19]. Furthermore, sedentary activities such as watching television, using phones, and playing video games are more common in urban areas [60]. Rural children have frequent PE classes [21] and more opportunities for outdoor play [19,21,61].

Children in Kenya, particularly urban girls, have low CRF, with only a small percentage meeting aerobic fitness threshold [30]. Boys tend to have better CRF than girls, consistent with previous studies in Kenya [25,36]. However, some of these studies may not accurately represent urban and rural children, and testing at high altitudes makes it challenging to determine their sea-level fitness [25,36]. Other studies show that girls have lower CRF than boys in urban and rural areas [62,63]. However, one US study found that urban children had better CRF levels than rural children [64]. These findings raise concerns about children’s health and cardiovascular disease risks. However, biological factors, socioeconomic disparities, and lifestyle choices could contribute to the observed differences in aerobic fitness [21,65].

The results indicate that urban children exhibited greater explosive leg power than their rural peers, with girls averaging less jump distance than boys. This finding aligns with a Macedonian study that supports the influence of environmental factors and places of residence on promoting PA and health [66]. Furthermore, the results concur with previous research suggesting that boys show superior performance in explosive leg power tests than girls [67] and another study that found boys from urban areas scored higher than their rural counterparts [67]. These variations likely arise from differences in resources, structured PA programmes, environmental factors, and opportunities for outdoor play, as children in rural areas engage in more physically demanding daily activities [68,69]

The findings also indicate that children from urban areas demonstrate superior hand grip strength compared to their rural counterparts, consistent with previous research showing that urban children with higher BMI tend to have stronger grip strength [25,30,70,71]. However, BMI alone does not fully explain these differences, as it does not account for muscle mass or neuromuscular coordination. Importantly, even after controlling BMI, urban–rural differences in grip strength persist, suggesting that other factors, such as volume or intensity of PA, lifestyle differences, or genetic variations, may also play a role.

While increased muscle mass associated with higher BMI may enhance grip strength, other factors such as muscle quality, neuromuscular coordination, and PA levels are critical. Environmental influences, including access to recreational facilities and community support, further shape these outcomes [52,72]. The results also indicate that girls typically have weaker hand grip strength than boys. While previous studies have suggested that measurement methods and socioeconomic status contribute to these differences [71], our study employed standardised evaluation across all participants. This standardisation implies that observed differences may stem from access to training resources, participation in physical activities, and physiological factors like muscle mass development and hormonal influences, rather than methodological discrepancies [52,73].

Although the current study found that urban children had stronger hand grip strength than rural children, other research suggests that rural children generally outperform urban children in grip strength [66,74]. Additionally, higher grip strength is associated with a lower likelihood of being overweight or obese [75]. The findings highlight the complex relationship between hand grip strength, BMI, and urban–rural disparities, emphasising the need for further research to understand these dynamics. Future studies should investigate the mechanisms behind these disparities, focusing on environmental, socioeconomic, and cultural factors. Additionally, localised data on PA patterns, access to recreational facilities, cultural norms, school PE quality, and dietary habits is crucial for developing targeted public health interventions that address both undernutrition in rural areas and overweight/obesity in urban settings.

### 4.1. Strengths and Limitations and Future Research

To our knowledge, this study is the largest and the first in nearly a decade to assess the fitness levels of Kenyan school children in urban and rural areas. The main strengths of this study lie in its ability to highlight significant public health concerns regarding health-related fitness disparities among children in Kenya. By involving a large and diverse sample from both urban and rural areas, the study provides valuable insights into these disparities and serves as a foundation for future research on physical fitness among children. It contributes to scientific knowledge by identifying differences in fitness levels, which are critical for shaping public health initiatives aimed at improving children’s well-being in low- and middle-income countries like Kenya. Additionally, the study builds on earlier research by addressing the pressing issues of childhood obesity and fitness, emphasizing the importance of up-to-date data in an evolving public health landscape. This evidence is crucial for guiding policy decisions to enhance the health and well-being of children in diverse environments

The study also has several limitations which must be acknowledged. While the findings may not generalize beyond the specific age group studied, they still offer a critical foundation for understanding fitness trends in this population. Another limitation was the lack of data on daily PA levels and nutritional status, which hindered insights into factors influencing PF. The lack of biological maturation and socioeconomic data is acknowledged, but the study’s rigorous methodology, including the use of motivated assistants to support participants, helped ensure the reliability of the test outcomes. Complete blinding was not feasible in this study due to the nature of the PF assessments. However, measures were taken to minimise potential bias. Additionally, although reference values were drawn from work validated among European and Australian populations, this highlights the urgent need for developing localised norms for African populations, which this study can help inspire. These contributions remain significant for advancing public health research and policy in similar contexts.

Future studies should focus on developing reference values for PF in children and adolescents in Sub-Saharan Africa. Also, future studies should include variables like biological maturation, dietary habits, and household income to better understand factors influencing children’s PF. Researchers should also gather detailed data on PA using devices such as accelerometers, alongside examining the impact of daily movement habits on children’s fitness. Although this study provides evidence on the fitness levels of children in Kenya, there is a need for further research to track trends in their PF.

### 4.2. Practical Implications

This study underscores the urgent need for targeted public health interventions to enhance PF among Kenyan children, addressing disparities between urban and rural areas. Kenya’s National Physical Activity Action Plan aims to promote active lifestyles, but struggles with funding, untrained staff, and low community engagement. Policies need strengthening to meet children’s needs and ensure effective monitoring. Urban initiatives should promote healthy eating and safe PA spaces, especially for girls, while rural efforts must combat undernutrition and improve access to nutritious foods alongside strength and endurance activities. Inclusive PA programmes for girls through gender-sensitive initiatives, female role models, equal access, and community engagement to challenge norms should be promoted. Comprehensive school programmes should leverage cultural practices and existing resources. Early interventions are vital to prevent chronic diseases associated with unhealthy weight, and the findings can inform national PF norms for tracking progress and evaluating effectiveness.

## 5. Conclusions

A higher percentage of children from urban areas are overweight or obese, while a higher percentage of children from rural areas are underweight, highlighting significant weight-related health challenges in both environments. Urban and rural children also have differences in fitness levels. Cardiorespiratory fitness is low among children, particularly girls in urban areas. The contrasting results for various aspects of PF highlight inherent inequities in the health-related PF and the correlates of urban and rural environments where children live and attend school as a strong determinant of fitness levels among Kenyan children. Additionally, the findings emphasise the need to assess children’s PA and movement behaviours to track and improve their fitness and the importance of customised and inclusive PA and fitness programmes for children in different rural and urban school environments. Finally, integrating Cohen’s d with univariate analysis results provides a comprehensive insight into health and fitness disparities, highlighting the need to consider multiple dimensions in future assessments.

## Figures and Tables

**Table 1:** Demographic and anthropometric characteristics of participants.

Variable	All (n = 1131)		Rural (n = 481)		Urban (n = 650)		*p*-Value	Cohen’s d
	Mean (SD) or n (%)		Mean (SD) or n (%)		Mean (SD) or n (%)			
Sex								
Boys	536 (47.4%)		232 (48.2%)		304 (46.8%)		0.55	
Girls	595 (52.6%)		249 (51.8%)		346 (53.2%)			
Age (years)	11.07	(0.89)	11.19	(0.95)	10.97	(0.83)	<0.001 *	0.25
Stature (cm)	138.80	8.92	135.45	7.46	141.27	9.10	<0.001 *	−0.70
Body mass (kg)	33.37	8.53	29.04	6.17	36.57	8.63	<0.001 *	−0.99
BMI (kg/m^2^)	17.15	3.31	15.72	2.36	18.21	3.51	<0.001 *	−0.83
Weight status								
Healthy weight	680 (60.1%)		245 (50.9%)		434 (66.9%)		<0.001 *	
Below healthy weight	303 (26.8%)		214 (44.5%)		89 (13.7%)			
Overweight	127 (11.2%)		19 (4%)		106 (16.6%)			
Obese	21 (1.9%)		3 (0.6%)		20 (2.8%)			
Type of school								
Private school	549 (48.5%)		197 (41%)		352 (54.2%)		<0.001 *	
Public school	582 (51.5%)		284 (59%)		298 (45.8%)			

* *p* < 0.05, compared rural and urban characteristics. Values are presented as mean (SD) for continuous variables and % for categorical variables. Abbreviations: BMI = body mass index; SD = standard deviation.

**Table 2:** Proportion of children achieving cardiorespiratory fitness (CRF), explosive leg power, and arm strength thresholds.

Variable	Rural			Urban			chi-Squared	df	*p*-Value
	n	%	Adjusted Residual	n	%	Adjusted Residual			
CRF Threshold (laps)	109	22.7%	6.7	55	8.4%	−6.7	45.102 ^a^	1	<0.001 *
Explosive Leg Power Threshold (cm)	382	79.4%	-	490	75.3%	-	2.691 ^a^	1	0.101
Dominant Arm Strength Threshold (kg)	320	66.5%	−8.2	565	86.8%	8.2	66.569 ^a^	1	<0.001 *

* *p* < 0.05, compared rural and urban children meeting thresholds for 20 m MSFT, SBJ, and hand grip. ^a^ indicates that the value represents the chi-squared test statistic between rural and urban children. Abbreviations: 20 m MSFT = twenty-meter multistage fitness test, SBJ = standing broad jump.

**Table 3:** Standardized coefficients for association between fitness variables and predictor variables.

Outcome Variable	Predictors	Multivariable Analysis				Univariate Analysis	
		Estimate (β)	Sig.	95.0% CI		Estimate (β)	Sig.
				LB	UB		
Speed, endurance, and coordination (number of bounces)	(Constant)	14.26	<0.001 *	8.72	19.80		
	Area (Urban)	3.68	<0.001 *	2.74	4.62	4.12	<0.001 *
	Sex (Female)	0.75	0.092	−0.12	1.62	0.98	0.045 *
	Age (years)	1.60	<0.001 *	1.10	2.09	1.85	<0.001 *
	Healthy Weight (reference)						
	Below Healthy Weight	−0.29	0.591	−1.34	0.76	−0.45	0.512
	Overweight	−5.83	<0.001 *	−7.24	−4.41	−6.12	<0.001 *
	Obese	−5.24	0.001*	−8.37	−2.10	−5.89	0.002 *
Lower back flexibility (cm)	(Constant)	20.81	<0.001 *	19.95	21.68		
	Sex (Female)	0.82	0.179	−0.38	2.02	1.12	0.145
	Area (Urban)	−2.77	<0.001 *	−3.91	−1.63	−3.13	<0.001 *
	Age (years)	4.19	0.063	−0.02	0.86	0.98	0.062
	Healthy Weight (reference)						
	Below Healthy Weight	0.32	0.511	−0.63	1.27	0.45	0.512
	Overweight	−1.18	0.073	−2.47	0.11	−3.12	0.073
	Obese	−0.46	0.745	−3.22	−2.30	−2.89	0.198
	Sex (female) × Area [urban-(reference)]						
	Sex (female) × Area (urban)	1.83	0.023 *	0.249	3.404	-	-
Hand grip strength (kg)	(Constant)	1.94	0.227	−1.21	5.10		
	Sex (Female)	−0.95	<0.001 *	−1.44	−0.45	−1.12	<0.001 *
	Area (Urban)	2.16	<0.001 *	1.62	2.69	2.45	<0.001 *
	Age (years)	1.39	<0.001 *	1.11	1.67	1.65	<0.001 *
	Healthy Weight (reference)						
	Below Healthy Weight	−1.98	<0.001 *	−2.59	−1.38	−2.12	<0.001 *
	Overweight	1.44	<0.001 *	.639	2.25	1.78	<0.001 *
	Obese	1.68	0.059	−0.06	3.41	2.01	0.045 *
Cardiorespiratory fitness (number of laps)	(Constant)	22.67	<0.001 *	21.30	24.05		
	Sex (Female)	−6.92	<0.001 *	−8.63	−5.21	−7.45	<0.001 *
	Area (Urban)	−4.67	<0.001 *	−6.37	−2.97	−5.12	<0.001 *
	Age (years)	0.10	0.760	−0.53	0.73	1.85	<0.001 *
	Healthy Weight (reference)						
	Below Healthy Weight	3.09	<0.001 *	1.75	4.44	3.45	<0.001 *
	Overweight	−5.03	<0.001 *	−6.84	−3.22	−5.45	<0.001 *
	Obese	−2.53	0.206	−6.45	1.39	−3.12	<0.001 *
	Sex(female) × Area [rural-(reference)]						
	Sex(female) × Area(urban)	3.00	0.009 *	0.76	5.25	-	-
Running speed and agility (s)	(Constant)	17.23	<0.001 *	16.97	17.49		
	Sex (Female)	0.49	<0.001 *	0.25	0.74	0.65	<0.001 *
	Area (Urban)	−1.03	<0.001 *	−1.30	−0.77	−1.25	<0.001 *
	Age (years)	−0.04	0.613	−0.18	−0.10	0.98	0.062
	Healthy Weight (reference)						
	Below Healthy Weight	0.14	0.342	−0.15	0.44	0.25	0.312
	Overweight	0.72	<0.001 *	0.32	1.12	0.85	<0.001 *
	Obese	0.92	0.036 *	0.06	1.79	1.12	0.045 *
Explosive leg power (cm)	(Constant)	101.81	<0.001 *	88.81	114.82		
	Sex (Female)	−6.75	<0.001 *	−8.77	−4.73	−7.12	<0.001 *
	Area (Urban)	2.25	0.046 *	0.04	4.46	2.78	0.035 *
	Age (years)	2.23	<0.001 *	1.08	3.38	2.45	<0.001 *
	Healthy Weight (reference)						
	Below Healthy Weight	−0.82	0.512	−3.29	1.64	−1.12	0.512
	Overweight	−12.22	<0.001 *	−15.54	−8.91	−13.12	<0.001 *
	Obese	−12.65	0.001 *	−19.84	−5.46	−13.45	0.002 *

* *p* < 0.05, association between fitness variables and predictor variables. Abbreviations: CI = confidence interval; LB = lower bound; UB = upper bound. β = unstandardized regression coefficient (β values represent estimated change in the outcome variable for a one-unit change in the predictor variable). Positive values indicate higher performance, while negative values indicate lower performance relative to the reference group.

## Data Availability

The data presented in this study are available on request from the corresponding author due to the fact that the study received ethical approval with the condition that only the research team would have access to participants’ data. The data collected in this study include personal information at both the individual and school levels, which could identify individuals. Participants did not give consent for their data to be made public. Those interested in accessing the data can request it from the Swansea University College of Engineering Research Ethics Committee at (coeresearchethics@swansea.ac.uk) and the Ethics and Scientific Review Committee at Kenyatta University (chairman.kuerc@ku.ac.ke).

## Abbreviations

PF	physical fitness
CRF	cardiorespiratory fitness
PA	physical activity
BMI	body mass index
Kenya-LINX	Kenya healthy diet and active lifestyle infrastructure for the next generation
KU-ERC	Kenyatta University Ethical Review Committee
NACOSTI	National Commission for Science, Technology, and Innovation
PE	physical education
20 m MSFT	20-metre multistage fitness test
cm	centimetres
kg	kilograms
IOTF	international obesity task force
SAR	sit-and-reach test
5 × 10 m SRT	5 × 10-metre shuttle run test
SBJ	standing broad jump test
s	second
m	metres
SD	standard deviation
χ^2^	Chi-square test
VIF	variance inflation factor
SPSS	Statistical Package for the Social Sciences
CI	confidence interval
LB	lower bound
UB	upper body
